# Pain, not chronic disease, is associated with the recurrence of depressive and anxiety disorders

**DOI:** 10.1186/1471-244X-14-187

**Published:** 2014-06-25

**Authors:** Marloes MJG Gerrits, Patricia van Oppen, Stephanie S Leone, Harm WJ van Marwijk, Henriëtte E van der Horst, Brenda W Penninx

**Affiliations:** 1 Department of Psychiatry, EMGO Institute for Health and Care Research, VU University Medical Center and Academic Outpatient Clinic for Affective Disorders, GGZ inGeest, Amsterdam, The Netherlands; 2 Department of General Practice and Elderly Care Medicine, EMGO Institute for Health and Care Research, VU University Medical Center, Van der Boechorststraat 7, 1081 BT Amsterdam, The Netherlands; 3 Department of Psychiatry, Leiden University Medical Center, Leiden, The Netherlands; 4 Department of Psychiatry, University Medical Center Groningen, Groningen, The Netherlands

**Keywords:** Depressive disorder, Anxiety disorder, Recurrence, Pain, Chronic diseases

## Abstract

**Background:**

Studies suggest that poor physical health might be associated with increased depression and anxiety recurrence. The objectives of this study were to determine whether specific chronic diseases and pain characteristics are associated with depression and anxiety recurrence and to examine whether such associations are mediated by subthreshold depressive or anxiety symptoms.

**Methods:**

1122 individuals with remitted depressive or anxiety disorder (Netherlands Study of Depression and Anxiety) were followed up for a period of four years. The impact of specific chronic diseases and pain characteristics on recurrence was assessed using Cox regression and mediation analyses.

**Results:**

Chronic diseases were not associated with recurrence. Neck (HR 1.45, p < .01), chest (HR 1.65, p < .01), abdominal (HR 1.52, p < .01) pain, an increase in the number of pain locations (HR 1.10, p < .01) and pain severity (HR 1.18, p = .01) were associated with an increased risk of depression recurrence but not anxiety. Subthreshold depressive symptoms mediated the associations between pain and depression recurrence.

**Conclusions:**

Pain, not chronic disease, increases the likelihood of depression recurrence, largely through its association with aggravated subthreshold depressive symptoms. These findings support the idea of the existence of a mutually reinforcing mechanism between pain and depression and are indicative of the importance of shedding light on neurobiological links in order to optimize pain and depression management.

## Background

Depressive and anxiety disorders are often recurrent. Recurrence rates of 25 to 60% have been reported in studies on different populations and using various methodologies [1-11]. After two to three years in remission, recurrence rates seem to stabilize after a person stays well for a longer period, the risk of recurrence is diminished [1,7,12]. Insight into risk factors for the recurrence of depressive and anxiety disorders is necessary in order to identify strategies to prevent new episodes as well as individuals who might benefit from long-term treatment [13].

Chronic diseases and/or pain might pose an important -potentially modifiable - risk for recurrence of depressive and anxiety disorders for various reasons [14]. Firstly, a negative impact for several chronic diseases and pain on the prevalence, the course of illness and treatment outcome for depression and anxiety was previously assessed [15-24]. Secondly, chronic diseases and pain can lead to negative coping strategies, disability and reduced quality of life [25,26], which might in turn lead to a further depressive or anxiety episode. Finally, shared pathophysiological mechanisms have been postulated for chronic somatic diseases and pain, on the one hand, and depression and anxiety, on the other [27-29]. So far, there is limited evidence for the effect of chronic diseases, especially specific diseases, and pain on depression and anxiety recurrence [30]. One study found that higher pain severity predicted depression relapse in 278 randomized trial subjects [31]. A treatment trial showed that cumulative chronic disease ratings significantly impacted depression relapse (n = 128) [32], whereas another found no effect for chronic conditions (n = 251) [8]. Two longitudinal studies (n = 585, n = 687 respectively) found that chronic diseases [3,4] and chronic pain [3] did not predict depression recurrence. One study (n = 429) found no effect for the number of chronic diseases on anxiety recurrence in a multivariate model [33]. To the best of our knowledge, there are no studies reporting associations between pain and anxiety recurrence. Previous studies on risk factors for recurrence of depressive and anxiety disorders point to subthreshold depressive and anxiety symptoms as the most consistent predictive factors for depression and anxiety recurrence [1-6,8-10,13,22,31,34-36]. Since chronic diseases and pain can also contribute to subthreshold depressive and anxiety symptoms [37,38], chronic diseases and pain and recurrence of depressive and anxiety disorders may be linked through higher subthreshold depressive and anxiety levels.

The objectives of this study were to examine to what extent chronic disease in general and specific diseases and pain symptoms are associated with recurrence of depressive and anxiety disorders in patients with a prior history of a depressive and/or anxiety disorder in a major longitudinal study. And second, to determine whether subthreshold depressive and anxiety symptoms partly mediate associations between chronic diseases and pain, on the one hand, and recurrence of depressive and anxiety disorders, on the other.

## Methods

### Sample

The Netherlands Study of Depression and Anxiety (NESDA) is a longitudinal cohort study, which was designed to examine the long-term course and consequences of depressive and anxiety disorders [39]. At baseline, 2981 participants (18 to 65 years), were included from the general population (n = 564), general practices (n = 1610) and mental health care organizations (n = 807). Exclusion criteria were not being fluent in Dutch and having a primary diagnosis of psychotic, obsessive compulsive, bipolar or severe addiction disorder. The Ethical Committee of the participating universities approved the research protocol and written informed consent was obtained from all patients. Baseline data collection took place between 2004 and 2007, with follow-up assessments, including the CIDI, two and four years later. The face-to-face interview assessments included a standardized diagnostic psychiatric interview and demographic and personal characteristics. Specially trained research staff conducted the interviews.

All 2981 participants were screened for depression and anxiety at baseline using the DSM-IV based Composite International Diagnostic Interview (CIDI, Version 2.1), a highly reliable and valid instrument for assessing depressive (major depressive disorder, dysthymia) and anxiety (social phobia, generalized anxiety disorder, panic disorder, agoraphobia) disorders [40]. Participants could have either no history, a prior history or a current depressive and/or anxiety disorder.

For this study, we examined 1236 participants who reported to have had a depressive or anxiety disorder in the past, but were currently in remission according to the CIDI, which meant they did not have a diagnosis of a depressive or anxiety disorder (in the previous six months) either at baseline (n = 628) or at the two year follow-up assessment (n = 608). Of these, 114 (9.2%), who did not differ significantly in age, gender, education or subthreshold depressive or anxiety symptoms at baseline, did not participate in the follow-up assessments. Consequently, a total of 1122 participants were followed up for up to four years.

### Recurrence of depressive and anxiety disorders

*Recurrence of a depressive or anxiety disorder* (yes/no) was defined by the DSM-IV based CIDI in the subsequent assessments. We calculated the *time to recurrence of a depressive or anxiety disorder* in months (maximum of 48 months) from the time the participant was assessed as being in remission (did not have a current diagnosis) until they were diagnosed with a depressive or anxiety disorder in one of the follow up assessments according to the CIDI. When a participant was diagnosed with a recurrence at the 2- or 4-year follow up assessment, participants were asked to indicate the recency of onset: less than a month ago, between one and 6 months ago, between 6 and 12 months ago, 12 months ago and between 12 and 24 months ago retrospectively. We used this information to calculate the median of the interval to recurrence. For instance, if a participant reported a recurrence of depressive or anxiety disorder between 1 and 6 months ago (median 3 months ago) at the 4-year follow up interview (and no disorder at the two year follow up), the time from baseline to recurrence to be used in the analyses was (48-3=) 45 months. For participants with no recurrence of depression or anxiety, time was censored as the time from the assessment in which remission was defined until the end of the follow up period.

### Measurements

Data on self-reported chronic diseases and pain, both indicators of physical health, were assessed at the point at which the participants were in remission (either at baseline or at the two-year follow up assessment).

#### ***Chronic disease***

First, patients were asked in a face-to-face interview whether they had been diagnosed with any of the chronic somatic diseases mentioned (Table 1). In order to provide the most objective assessment of chronic diseases, we only deemed chronic disease to be present if the participant stated that the disease was being treated by a healthcare professional or he or she was using medication for the disease. As in a previous study [18], we classified chronic diseases into seven main categories; cardiometabolic, respiratory, endocrine, neurological, musculoskeletal, digestive disorders and cancer. We defined the number of chronic diseases as the number of disease categories into which a participant fell.

**Table 1:** Sample characteristics

**Characteristics**	**Population N = 1122 (%)**
**Sample characteristics**	
Female gender,%	765 (68.2%)
Age in years, Mean (SD)	43.4 (12.8)
Education in years, Mean (SD)	12.5 (3.2)
Recency of last episode (≤1 year),%	160 (14.3%)
History of both depression and anxiety,%	543 (48.4%)
QIDS score, Mean (SD)	5.4 (3.7)
BAI, Mean (SD)	7.2 (6.4)
**Physical health characteristics**	
Chronic disease category,%	
*Cardiometabolic*	
Hypertension, angina pectoris, history of cardiac disease, stroke, diabetes	185 (16.5%)
*Respiratory*	
Asthma, chronic bronchitis, pulmonary emphysema	88 (7.8%)
*Musculoskeletal*	
Osteoarthritis, rheumatoid arthritis, systemic lupus, erythematodes, fibromyalgia	112 (10.0%)
*Digestive*	
Ulcer, irritable bowel syndrome, Crohn’s disease, colitis, ulcerosa, diverticulitis, liver cirrhosis, hepatitis, constipation	101 (9.0%)
*Neurological*	
Migraine, epilepsy, multiple sclerosis, peripheral, neuropathy, hernia	48 (4.3%)
*Endocrine*	
Thyroid dysfunction	36 (3.2%)
*Cancer*	
Throat, thyroid, lymphoid, lung, esophagus, bowel, stomach, liver, uterus, cervix, ovary, bladder, testicle, prostate, skin, brain, blood	83 (7.4%)
Number of chronic diseases, Mean (SD)	0.6 (0.8)
Pain location,%^1^	
Neck	238 (21.2%)
Back	297 (26.5%)
Head	288 (25.7%)
Orofacial	59 (5.3%)
Abdominal	189 (16.8%)
Joints	245 (21.8%)
Chest	80 (7.1%)
Number of pain locations, Mean (SD)^1^	1.2 (1.9)
Duration of Pain ≥ 90 days,%	355 (31.6%)
Chronic Pain Grade (CPG)	
CPG 0-1	720 (64.2%)
CPG 2	246 (21.9%)
CPG 3	101 (9.0%)
CPG 4	54 (4.8%)
**Outcome,%**	
Recurrence of depression disorder	292 (26.0%)
Recurrence of anxiety disorder	255 (22.7%)
Recurrence of depressive and/or anxiety disorder	424 (37.8%)
Duration of follow up in months, Mean (SD)	30.0 (13.4)

^1^Pain locations were only taken into account when CPG ≥ 2.

*Abbreviations*: *CPG* Chronic Pain Grade, *QIDS* Quick Inventory of Depressive Symptoms, *BAI* Beck Anxiety Inventory.

#### ***Pain***

To assess pain over the last 6 months, the interview contained four different items: a) 7 specific common pain locations (neck, back, head, orofacial area, abdomen, chest and joints); b) the number of locations; c) duration and; d) severity determined by the Chronic Pain Grade (CPG) [41]. First, the number of pain locations (0–7) in the last six months was assessed. Then, the participant was asked to choose the most painful site, to which all subsequent questions applied. Next, duration of pain in the last six months was dichotomized as ≥90 versus <90 days, based on the most frequently used definition of chronic pain. Last, severity was graded by measuring the intensity of pain and disability caused by pain using the CPG scale:

– Grade 1: low intensity-low disability

– Grade 2: high intensity-low disability

– Grade 3: high disability-moderately limiting

– Grade 4: high disability-severely limiting

To exclude mild and sporadic pain symptoms experienced in the past six months, only pain locations with at least a Grade 2 on the CPG were taken into account. Only the more severe pain locations (at least high intensity of pain and low disability caused by pain) were included.

#### ***Covariates***

Sociodemographic characteristics included age, gender and number of years in education. The recency of the last episode of depressive or anxiety disorder, more than one year ago or one year or less, was determined at the time participants were deemed to be in remission from a depressive or anxiety disorder, since the time from remission might impact on the recurrence of depression and anxiety [1]. Additionally, to account for possible psychoactive and pain medication effects, medication use was assessed based on drug container inspection of all drugs used in the month prior to the interview and classified according to the World Health Organization Anatomical Therapeutic Chemical (ATC) classification. Antidepressants included selective serotonin reuptake inhibitors (ATC-code N06AB), tricyclic antidepressants (N06AA) and other antidepressants (N06AF/N06AX). Benzodiazepines included ATC-codes N03AE, N05BA, N05CD and N05CF. Pain medication included paracetamol (N02BE01), acetylsalicylic acid (N02BA), non-steroidal anti-inflammatory drugs (M01A, M01B), and opioids (N02A).

#### ***Mediators***

A mediator is an intervening variable that may account for the association between the independent variables, chronic disease and pain, and the dependent variable, the recurrence of depression or anxiety. We assessed the subthreshold symptoms of depression and anxiety using the severity of symptoms at the time participants were deemed to be in remission from depressive or anxiety disorders. We expected subthreshold symptoms to be partial mediators since the presence of chronic diseases and pain have been shown to increase severity of symptoms of depression and anxiety [37,38], which could in turn lead to depression or anxiety recurrence [1-3,5,6,8,10,31,34,35]. The severity of depressive symptoms was measured using the Quick Inventory of Depressive Symptomatology -self-report (QIDS), a reliable and valid instrument consisting of 16 items (0–27 score) [42,43]. Self-reported anxiety symptom severity was measured using the reliable and valid Beck Anxiety Inventory (BAI), consisting of 21 items (0–63 score), measuring severity of mainly arousal-related symptoms of anxiety [44].

### Statistical analysis

To examine the associations of chronic diseases and pain with the time to recurrence of a diagnosis of depression or anxiety, we performed Cox regression analyses, before and after adjustment for covariates. Cox regression takes into account differences in time at risk for an event and censoring. Time at risk was measured from the moment the participant was assessed as being in remission until either the participant had an event, a recurrent depressive or anxiety disorder, or was censored when the participant did not have a recurrence during the follow up period. We also analyzed the time to recurrence of depressive and anxiety disorder separately, in order to explore whether the impact of chronic diseases and pain is different for recurrence of depression versus anxiety.

**Figure 1:**
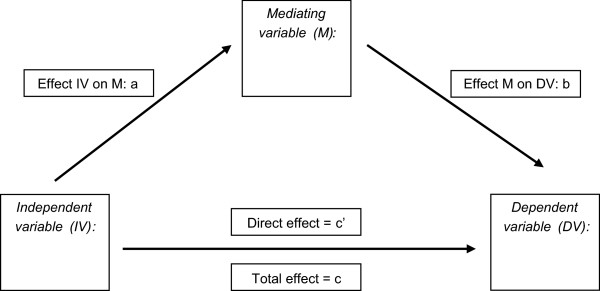
**Mediation model.** A = effect of independent variable on mediating variable. B = effect of mediating variable on dependent outcome. C = direct effect of independent variable on dependent variable. C’ = total effect of independent variable on dependent variable. AxB = indirect effect of independen variable on dependent variable through mediating variable.

To determine whether subthreshold symptoms of depression and anxiety mediated the associations found between chronic diseases, pain and recurrence of depression or anxiety, we conducted mediation analyses, through the indirect method by Preacher and Hayes involving bootstrapping approximations (Figure 1) [45].

As shown in the model below, the indirect method estimates the total, direct, and indirect unstandardized effects of the independent variables on the dependent variable through the mediator variable, controlling for covariates. The results will show the effect “a” of the independent variable (pain variable) on the mediating variable (depression severity), and the effect “b” of the mediating variable on the dependent outcome (depression recurrence). The effect of the pain variables on the recurrence of a depressive disorder will be shown by the direct “c’” effect. The effect of the pain variables on the recurrence of a depressive disorder through depression severity will be shown by the indirect “a × b” and total “c” effect”.

## Results

### Sample characteristics

Table 1 shows the characteristics of the study sample (n = 1122). Mean age of the study sample was 43.4 years and 68.2% were female. The average number of chronic diseases was 0.6 (SD 0.8), 59.4% of participants had no chronic disease. The mean number of pain locations was 1.2 (SD 1.9) and 35.8% had a CPG of at least 2. Of the participants, 424 (37.8%) experienced a recurrence of a depressive and/or anxiety disorder during the follow-up period. Of participants with a least one chronic disease 39.1% had a recurrence, whereas participants with severe or disabling pain (CPG > =2) 42.4% had a recurrence. When considering depression and anxiety recurrence separately, 26.0% and 22.7% participants had a recurrence, respectively. For depression recurrence, participants with severe or disabling pain (CPG > =2) were more likely to experience a recurrence; 30.9%. The average follow-up period was 30.0 months (SD 13.4).

### Impact of chronic diseases and pain on recurrence

Table 2 shows the adjusted associations between chronic diseases, pain symptoms and recurrence of a depressive or anxiety disorder. Unadjusted analyses showed similar results (not shown). For the chronic disease categories no associations were found for recurrence of depression or anxiety. Significant positive associations were found with recurrence of depression and/or anxiety. However, such associations were solely driven by the recurrence of depressive disorder, not anxiety. For instance, neck pain was significantly associated with 45% increased risk for depression recurrence (HR 1.45, p = .005). Similarly, chest (HR = 1.65, p = .008) and abdominal (HR = 1.52, p = .003) pain were significantly associated with depression recurrence, as was the number of pain locations (per location increase: HR = 1.10, p = .002) and higher CPG (per grade increase: HR = 1.18, p = .01). Additionally adjusting for use of psychotropic or pain medication did not alter the results.

**Table 2:** **Associations between somatic health variables and time to recurrence of depressive and/or anxiety disorder during follow-up (n = 1122)**^**a**^

**Physical health characteristics**	**Time to recurrence of depressive and/or anxiety disorder**^**c**^		**Time to recurrence of depressive disorder**^**c**^		**Time to recurrence of anxiety disorder**^**c**^	
	**HR (95% CI)**	**p**	**HR (95% CI)**	**p**	**HR (95% CI)**	**p**
*Chronic disease category*						
Cardiometabolic	0.94 (0.71-1.25)	.66	0.86 (0.61-1.21)	.38	1.11 (0.78-1.59)	.57
Respiratory	1.17 (0.84-1.65)	.36	1.31 (0.89-1.93)	.18	1.10 (0.70-1.71)	.69
Musculoskeletal	1.13 (0.83-1.55)	.44	1.35 (0.94-1.92)	.11	1.19 (0.80-1.77)	.39
Digestive	1.26 (0.92-1.73)	.15	1.19 (0.82-1.75)	.36	1.22 (0.81-1.84)	.34
Neurological	1.17 (0.74-1.86)	.50	1.22 (0.71-2.09)	.47	0.89 (0.46-1.74)	.74
Endocrine	1.09 (0.65-1.83)	.76	1.07 (0.57-2.02)	.84	1.53 (0.85-2.75)	.15
Cancer	1.13 (0.80-1.60)	.49	1.11 (0.73-1.68)	.62	1.21 (0.78-1.86)	.40
Number of chronic diseases	1.09 (0.97-1.23)	.16	1.11 (0.96-1.27)	.16	1.13 (0.97-1.31)	.12
*Pain location*^b^						
Neck	1.31 (1.05-1.64)	.02	1.45 (1.12-1.89)	.005	1.15 (0.86-1.55)	.35
Back	1.15 (0.93-1.43)	.21	1.30 (1.00-1.67)	.05	0.93 (0.70-1.24)	.65
Head	1.21 (0.98-1.50)	.08	1.29 (1.00-1.67)	.05	1.26 (0.95-1.66)	.11
Orofacial	1.34 (0.92-1.96)	.13	1.46 (0.94-2.27)	.09	1.20 (0.72-2.00)	.49
Chest	1.51 (1.09-2.08)	.01	1.65 (1.14-2.39)	.008	1.41 (0.93-2.14)	.11c
Abdominal	1.33 (1.05-1.68)	.02	1.52 (1.16-2.02)	.003	1.13 (0.82-1.55)	.45
Joints	1.20 (0.96-1.51)	.12	1.31 (1.00-1.71)	.05	0.99 (0.73-1.34)	.94
Number of pain locations^b^	1.07 (1.02-1.12)	.009	1.10 (1.04-1.16)	.002	1.03 (0.97-1.10)	.34
Duration of pain ≥ 90 days	1.10 (0.89-1.35)	.38	1.24 (0.96-1.58)	.11	0.99 (0.75-1.30)	.94
Chronic Pain Grade	1.11 (0.99-1.24)	.06	1.18 (1.04-1.35)	.01	1.07 (0.93-1.24)	.35

^a^Using univariate cox regression analyses.

^b^Pain locations were only taken into account when CPG ≥ 2.

^c^Adjusted for age, gender, years of education and recency of last episode of depressive or anxiety disorder.

### Mediation of subthreshold depressive and anxiety symptoms

In Table 3, we show the mediation of subthreshold depressive symptoms on the associations between pain and recurrence of depression which were analysed as borderline significant or significant in the Cox regression analyses (p < .10). We did not conduct these analyses for chronic diseases or recurrence of anxiety, since we did not find any significant associations for these indicators in initial analyses. First, mediation analyses confirmed that pain is associated with higher severity of subthreshold symptoms (a in Table 3) and that a higher severity of subthreshold symptoms was significantly associated with depression recurrence (b in Table 3). For all pain variables, the direct paths were assessed as insignificant, although the direct paths of abdominal and chest pain remained borderline significant (c’ in Table 3). The indirect effects (a × b in Table 3) were significant for pain of the neck, back, head, abdomen, chest and joints, higher number of pain locations and higher CPG, suggesting that there is an overall effect of the pain variables on depression recurrence through aggravated subthreshold depressive symptoms.

**Table 3:** **Summary of Preacher and Hayes mediator model analyses (5000 bootstraps) between pain (IV), and recurrence of depressive disorder (DV) through subthreshold depression severity as measured by the QIDS (M)**^**2**^

**Pain variables (IV)**	**Mediating variable (M)**	**Dependent variable (DV)**	**Effect of IV on M (a)**	**Effect of M on DV (b)**	**Direct effect (c’)**		**Indirect effect (a x b)**	**95% CI (ab)**	**Total effect (c)**	
					**Effect**	**p**			**Effect**	**p**
Neck^1^	Subthreshold depression severity	Recurrence of depression	1.55**	.16**	.25	.14	.25	(.14-.36)^	.47	.004
Back^1^			1.40**	.16**	.05	.76	.23	(.13-.34)^	.27	.09
Head^1^			1.25**	.16**	.12	.48	.20	(.11-.31)^	.30	.05
Abdominal^1^			1.79**	.16**	.35	.06	.28	(.17-.41)^	.60	.001
Chest^1^			2.51**	.16**	.43	.10	.40	(.23-.59)^	.77	.002
Joints^1^			1.21**	.16**	.16	.37	.19	(.10-.30)^	.34	.04
Number of pain locations^1^			.42**	.15**	.06	.12	.07	(.04-.09)^	.12	.001
Chronic Pain Grade			.74**	.16**	.08	.36	.12	(.07-.18)^	.18	.02

*p < .05, **p < .001, ^ significant based on 95% confidence interval (CI).

^1^Pain location was only taken into account when CPG ≥ 2.

^2^Adjusted for age, gender, years of education and recency of last episode of depressive and/or anxiety disorder.

## Discussion

The purpose of this study was to examine whether chronic diseases and pain are associated with recurrence of depressive and anxiety disorders and if so, whether these associations are partly mediated by subthreshold depressive and anxiety symptoms. In this large study with a mean follow up of 2,5 years, 37.8% of people experienced a recurrence, which corresponds reasonably well to recurrence rates found in previous research [1-11]. During follow-up a quarter of all participants had a depression recurrence, while a third of participants with severe or disabling pain had a recurrence. We did not detect an association between chronic disease and recurrence of depressive and anxiety disorders. Severe neck, chest and abdominal pain, a higher number of pain locations and higher pain severity of pain were significantly associated with recurrence of depressive disorder, but not anxiety. Subthreshold depressive symptoms mediated the associations between pain and depression recurrence.

The finding that chronic diseases are not associated with the recurrence of depression or anxiety in this study is in line with previous studies [3,4,8,33]. Iosifescu et al. found that increasing cumulative chronic disease ratings did predict relapse in a treatment trial [32]. Contrary to the measurements used by Iosifescu et al., we had no information on the severity of the chronic diseases and our study was based on a relatively healthy population with on average 0.6 chronic diseases. Also, the present study and the above mentioned studies investigated rather young populations with mean age around forty years [3,4,8,32]. Chronic diseases often progress over time so associations with depression and anxiety recurrence might be different in studies examining older multi-morbid populations.

This is the first observational study to systematically examine the associations between pain symptoms and depression and anxiety recurrence. We found that several pain characteristics are associated with a higher risk of depression recurrence, which concurs with results of Fava et al. who found that higher levels of pain severity predicted relapse of depression in trial subjects [31]. Since all pain locations were found to be borderline significant or significant, the particular location of the pain seems less relevant in predicting depressive disorder recurrence. Our results point to a dose–response relationship, where increasing number of pain locations and increasing pain severity seem to heighten the risk of depression recurrence. Our findings, with subthreshold depression levels largely mediating the associations, indicate that a mutually reinforcing mechanism between pain and depression might exist [27]. In support of such relationship, another study found that change in pain was a strong predictor of subsequent depression symptom severity and vice versa [46]. A vicious circle could arise with negative coping strategies in response to impaired physical and social role functioning caused by both the pain and affective symptoms [47,48]. Furthermore, evidence for various shared pathophysiological pathways has been found. Neuroimaging studies have shown overlap of the neuronal networks of emotion and pain, particularly in the (pre)frontal cortical regions [49]. Pain may cause changes such as dysregulation of the HPA axis (increasing cortisol levels), and of the autonomic nervous system (increased sympathetic or decreased parasympathetic function) leading to a new depressive disorder episode. It might also be possible that through these same pathways, previous depressive episode(s) have made patients more vulnerable to pain [22,27,47,50-52].

Contrary to what we expected, we did not find a significant relationship between pain and anxiety recurrence. Although the same pathophysiological pathways for depression and pain are mentioned in research on anxiety and pain, associations might be different or they might be linked through depressive disorders. For instance, HPA-axis abnormalities were found to be strongly associated with depressive disorder and co-morbid depression and anxiety, but were not present for most types of anxiety disorders [52,53]. A cross-sectional study found that depression partly mediated the relationship between pain and anxiety [21]. A previous NESDA study found that anxiety predicted the recurrence of anxiety only, whereas depression predicted the recurrence of both depression and anxiety [54]. Since research on anxiety and co-morbid depressive and anxiety disorders is still limited compared with depressive disorder alone, more research, particularly on anxiety, is needed to further explore such links.

This study shows that pain makes a patient vulnerable to recurrence of depressive disorders. Pain is among the most common reasons to consult a physician, so patients with pain who also have a depressive history are very likely to contact their physician. The physician then has the opportunity to enquire after depressive symptoms and start treatment, if necessary. Previous studies have shown that pain negatively impacts depression treatment and vice versa [27,55]. The current study suggests that it may be of benefit to prolong depression treatment until full remission, especially for those patients who also experience severe pain symptoms, as they are a risk group to remit into full-blown disorders. Also, specific relapse prevention trials could focus on distinctions between patients with and without pain in order to find new strategies to prevent depression relapse in patients with pain. Some evidence has been found for alleviating chronic pain and depressive symptoms with collaborative care initiatives for chronic pain patients [56,57]. Collaborative care trials have proven their efficacy on sustained recovery of depressive disorder and continuation and maintenance therapies can reduce recurrence [58,59]. Tailored collaborative care interventions with maintenance therapies for patients who recovered from a depressive disorder but who are experiencing pain might reduce recurrence risk in this particular subpopulation of vulnerable patients. Based on ever growing evidence on shared neurobiological pathways new effective interventions need to be explored.

Strengths of our study are the large sample size, the prospective design and the systematic investigation of the role of various chronic diseases and pain on the recurrence of both depression and anxiety. Also, we used full diagnostic interviews to assess the presence of depressive and anxiety disorders with questions on the recency of episodes which enabled us to assess time to recurrence more accurately. This study also had some limitations. The prevalences of specific chronic diseases (myocardial infarction, epilepsy etc.) were quite low, so our capacity to study the role of all diseases individually was limited. This study was conducted with patients of 18 to 65 years old; findings could be different in populations with younger individuals or elderly people. We analysed depressive and anxiety disorders separately, but did not analyse differences between specific depressive or anxiety disorders. The mediating variable, depressive symptoms, may partly measure the same construct as the outcome variable. The final limitation is that the statistical mediation model assumes temporal direction of the independent variable preceding the mediator, which precedes the outcome variable, but both pain and subthreshold symptoms were measured at the same assessment.

## Conclusions

The presence of chronic diseases does not increase the risk of recurrence of depressive or anxiety disorders. Pain increases the likelihood of the recurrence of depressive disorder. Since pain was associated with increased subthreshold depressive symptoms, this largely mediated the effects. Mutually reinforcing neurobiological mechanisms between pain and depressive disorders are likely to exist, which might be addressed in future treatment options, to prevent recurrences.

## Competing interests

The authors declare that they have no competing interests.

## Authors’ contributions

BP contributed to the conception and design of the study. MG and SL undertook the analyses and interpretation of data. MG made the first draft of the article, and BP, HM, SL, PO and HH contributed to revising the article critically for important intellectual content. All authors contributed to and have approved the final manuscript.

## Pre-publication history

The pre-publication history for this paper can be accessed here:

http://www.biomedcentral.com/1471-244X/14/187/prepub
